# IPSS-R及WPSS移植前评估对异基因造血干细胞移植治疗骨髓增生异常综合征患者预后意义的研究

**DOI:** 10.3760/cma.j.issn.0253-2727.2022.03.011

**Published:** 2022-03

**Authors:** 棽琦 陆, 畅 侯, 鹏 王, 露巍 张, 祎 范, 德沛 吴, 杨 徐

**Affiliations:** 苏州大学附属第一医院，国家血液系统疾病临床医学研究中心，江苏省血液研究所，国家卫生健康委血栓与止血重点实验室，苏州大学造血干细胞移植研究所，苏州 215006 The First Affiliated Hospital of Soochow University, National Clinical Research Center for Hematologic Diseases, Jiangsu Institute of Hematology, Key Laboratory of Thrombosis and Hemostasis Under Ministry of Health Institute of Blood and Marrow Transplantation, Soochow University, Suzhou 215006, China

**Keywords:** 骨髓增生异常综合征, 异基因造血干细胞移植, IPSS-R, WPSS, Myelodysplastic syndrome, Allogeneic hematopoietic stem cell transplantation, IPSS-R, WPSS

## Abstract

**目的:**

探究不同时间进行修订版国际预后积分系统（IPSS-R）及WHO预后积分系统（WPSS）评分对异基因造血干细胞移植（allo-HSCT）治疗骨髓增生异常综合征（MDS）患者的预后评估价值。

**方法:**

回顾性分析2016年7月至2019年6月于苏州大学附属第一医院行allo-HSCT的184例MDS患者临床资料，在初诊及移植前两个时间点进行IPSS-R及WPSS评分，比较两者预后评估价值，分析影响移植预后的危险因素。

**结果:**

中位随访21.9（0.5～47.5）个月，移植后2年总体生存（OS）、无进展生存（PFS）率分别为（75.1±3.4）％、（71.6±3.6）％，2年复发（RR）率和无复发死亡率（NRM）分别为（11.9±0.1）％、（16.5±0.1）％。初诊时IPSS-R≤3.5分与>3.5分组OS、PFS差异无统计学意义（*P*＝0.409；*P*＝0.724），WPSS≤2分与>2分组OS、PFS差异无统计学意义（*P*＝0.462；*P*＝0.726）。移植前除外10例患者转化为急性髓系白血病（AML），174例患者可进行IPSS-R及WPSS评估，其中IPSS-R≤3.5分组OS、PFS率显著优于>3.5分组［OS：（88.6±4.1）％对（65.8±5.3）％，*P*＝0.003；PFS：（87.6±4.2）％对（60.5±5.8）％，*P*＝0.002］，WPSS≤2分与>2分组间OS、PFS差异无统计学意义（*P*＝0.584；*P*＝0.565）。此外，移植前IPSS-R较初诊时相比改善组OS、PFS率显著优于未改善组［OS：（83.8±4.6）％对（69.3±5.8）％，*P*＝0.027；PFS：（82.8±4.4）％对（64.0±7.2）％，*P*＝0.006］，多因素分析发现移植前TP53突变（*P*＝0.047，*HR*＝2.460，95％ *CI* 1.014～5.971）和移植前IPSS-R>3.5分（*P*＝0.021，*HR*＝2.510，95％ *CI* 1.151～5.476）是影响OS的独立不良预后因素，移植前细胞遗传学差及极差（*P*＝0.008，*HR*＝2.765，95％ *CI* 1.305～5.856）和移植前IPSS-R>3.5分（*P*＝0.017，*HR*＝2.457，95％ *CI* 1.175～5.141）是影响PFS的独立不良预后因素。

**结论:**

移植前进行IPSS-R评分对行allo-HSCT的MDS患者的预后评估有重要价值，移植前IPSS-R较初诊时改善的患者有着更好的预后。

## Abstract (en)

**Objective:**

This study aimed to explore the prognostic value of the revised international prognostic scoring system (IPSS-R) and the WHO prognostic scoring system (WPSS) in patients with myelodysplastic syndrome (MDS) undergoing allogeneic hematopoietic stem cell transplantation (allo-HSCT).

**Methods:**

The clinical data of 184 patients with MDS who received allo-HSCT from July 2016 to June 2019 were retrospectively analyzed. IPSS-R and WPSS were performed at diagnosis and before transplantation. The prognostic values of IPSS-R and WPSS and potential risk factors were explored.

**Results:**

With a median follow-up of 21.9 (0.5–47.5) months, the two-year overall survival (OS) and progression-free survival (PFS) rates were (75.1±3.4)％ and (71.6±3.6)％, respectively. The two-year cumulative relapse rate and nonrelapse mortality rate were (11.9±0.1)％ and (16.5±0.1)％, respectively. There were no significant differences in OS and PFS between the IPSS-R ≤3.5 and >3.5 groups at diagnosis (*P*＝0.409; *P*＝0.724). No significant differences in OS and PFS between the WPSS ≤2 and >2 groups (*P*＝0.426; *P*＝0.726) were observed as well. When the patients were reevaluated before transplantation, the OS and PFS of the IPSS-R ≤3.5 group were significantly better than >3.5 group［OS: (88.6±4.1)％ *vs* (65.8±5.3)％, *P*＝0.003; PFS: (87.6±4.2)％ *vs* (60.5±5.8)％, *P*＝0.002］. However, there were no significant differences in OS and PFS among the WPSS ≤2 and >2 groups (*P*＝0.584; *P*＝0.565). In addition, the OS and PFS of the improved group based on IPSS-R were significantly better than those of the unimproved group before transplantation［OS: (83.8±4.6)％ *vs* (69.3±5.8)％, *P*＝0.027; PFS: (82.8±4.4)％ *vs*. (64.0±7.2)％, *P*＝0.006］. Multivariate analysis indicated that a pretransplant IPSS-R of >3.5 (*P*＝0.021, *HR*＝2.510, 95％ *CI* 1.151–5.476) and TP53 mutation (*P*＝0.047, *HR*＝2.460, 95％ *CI* 1.014–5.971) were independent risk factors for OS, whereas a pretransplant IPSS-R of >3.5 (*P*＝0.017, *HR*＝2.457, 95％ *CI* 1.175–5.141) and pretransplant cytogenetic poor and very poor (*P*＝0.008, *HR*＝2.765, 95％ *CI* 1.305–5.856) were independent risk factors for PFS.

**Conclusion:**

A pretransplantation evaluation of IPSS-R could help determine the prognosis of patients with MDS undergoing allo-HSCT. In addition, patients with improved IPSS-R scores before undergoing allo-HSCT had a better prognosis.

骨髓增生异常综合征（MDS）是一组异质性的髓系克隆性疾病，表现为病态造血或无效造血、高风险向急性髓系白血病（AML）转化[1]。MDS患者预后差异较大，通过进行准确的危险分层有助于制定合适治疗方案。目前广泛应用于临床的MDS预后评估系统包括国际预后积分系统（IPSS）、修订版国际预后积分系统（IPSS-R）及WHO预后积分系统（WPSS）[2]–[4]，其中IPSS适用于原发初诊的MDS患者，IPSS-R、WPSS可以动态监测患者预后[5]–[6]。异基因造血干细胞移植（allo-HSCT）是迄今为止唯一可能治愈MDS的治疗方案[7]，目前关于IPSS-R及WPSS评估移植患者预后的研究较少[5],[8]–[10]。本研究通过对苏州大学附属第一医院收治的184例接受allo-HSCT的MDS患者初诊及移植前进行IPSS-R及WPSS的评估，探究IPSS-R及WPSS在MDS移植患者预后评估中的价值。

## 病例与方法

1. 病例：以2016年7月至2019年6月在苏州大学附属第一医院接受allo-HSCT治疗的184例MDS患者为研究对象。MDS诊断符合《骨髓增生异常综合征中国诊断与治疗指南（2019年版）》[11]。根据WHO（2016）诊断标准，MDS伴有单系发育异常（MDS-SLD）6例、MDS伴环状铁粒幼红细胞（MDS-RS）2例、MDS伴有多系发育异常（MDS-MLD）35例、MDS伴有原始细胞过多-1（MDS-EB1）55例、MDS伴有原始细胞过多-2（MDS-EB2）84例、MDS未分类（MDS-U）2例。分别在初诊及移植前对患者进行IPSS-R及WPSS预后评分。

2. NGS检测：提取初诊患者骨髓单个核细胞，抽提基因组DNA，构建51个血液病相关的常见热点基因Ion AmpliSeq文库，使用ABI Ion Torrent S5测序仪进行检测。NGS扩增子平均基因覆盖率98.03％，平均测序深度2 500×，95％以上的目标区域测序深度大于2 000×。

3. 移植前治疗：59例患者移植前接受去甲基化药物（HMA）治疗，6例接受化疗，88例接受HMA联合预激治疗，预激方案包括CAG（阿克拉霉素、阿糖胞苷、G-CSF）、IAG（去甲柔红霉素+阿糖胞苷+G-CSF）、HAG（高三尖杉酯碱+阿糖胞苷+G-CSF）等，中位治疗2（1～5）个疗程，40例仅接受支持治疗。移植前完全缓解（CR）123例，部分缓解（PR）10例，疾病稳定（SD）17例，疾病进展（PD）24例，转化为AML 10例。

4. 供者及干细胞来源：亲缘供者156例，无关供者28例；供受者HLA全相合81例，其中亲缘全相合53例，无关全相合28例；供受者HLA不全相合103例。68例接受外周血干细胞移植（PBSCT），109例接受骨髓和外周血混合移植（BMT+PBSCT），7例接受骨髓移植（BMT）。回输单个核细胞细胞中位数9.8（1.7～20.4）×10^8^/kg，回输CD34^+^细胞中位数3.8（1.6～23.9）×10^6^/kg。

5. 预处理方案：168例患者采用清髓性预处理（MAC）方案，具体方案为改良BuCy：白消安0.8 mg/kg，每6 h 1次，−7～−5 d；环磷酰胺1.8 g·m^−2^·d^−1^，−4、−3 d。16例采用减低剂量预处理（RIC）方案，具体为FB方案：氟达拉滨30 mg·m^−2^·d^−1^，−7～−2 d；白消安3.2 mg·m^−2^·d^−1^，−7、−6 d。

6. 移植物抗宿主病（GVHD）防治：同胞全相合移植的患者采用甲氨蝶呤（MTX）15 mg/m^2^、+1 d，10 mg/m^2^、+3、+6 d；环孢素A（CsA）3 mg·kg^−1^·d^−1^，−1 d开始预防GVHD，无关全相合移植和单倍型移植的患者采用抗胸腺细胞球蛋白（ATG）、MTX、CsA及霉酚酸酯（MMF）预防GVHD。无关全相合移植GVHD预防方案：ATG 2.5 mg·kg^−1^·d^−1^，−4 ～−2 d；MTX 15 mg·m^−2^·d^−1^，+1 d，10 mg·m^−2^·d^−1^，+3、+6、+11 d；CsA 3 mg·kg^−1^·d^−1^，−8 d开始；MMF 15 mg·kg^−1^·d^−1^，−8 d开始。单倍型移植GVHD预防方案：ATG 2.5 mg·kg^−1^·d^−1^，−5～−2 d；MTX 15 mg·m^−2^·d^−1^，+1 d，10 mg·m^−2^·d^−1^，+3、+6、+11 d；CsA 3 mg·kg^−1^·d^−1^，−9 d开始；MMF 1.0 g/d，−9 d开始。

7. 相关标准和定义：中性粒细胞计数连续3 d>0.5×10^9^/L的首日定义为粒细胞植活时间，血小板计数连续7 d>20×10^9^/L且脱离血小板输注的首日为血小板植活时间。急性GVHD（aGVHD）及慢性GVHD（cGVHD）根据美国西雅图标准进行诊断及分级，cGVHD分为局限型（50％以下的皮肤受累或仅有肝脏受累）和广泛型cGVHD（累积任何其他器官，50％以上皮肤受累或肝硬化）。

8. 随访及疗效评定：随访时间截至2020年5月31日，通过病历及电话进行随访。总生存（OS）时间定义为干细胞回输至死亡或末次随访日，无进展生存（PFS）时间定义为干细胞回输至血液学进展、死亡或末次随访日。复发（RR）时间定义为从干细胞回输到血液学复发，非复发死亡时间从干细胞回输到非复发死亡或末次随访日。

9. 统计学处理：应用SPSS 25.0、RStudio进行数据分析，应用Graphpad Prism 8.0绘图。采用Kaplan-Meier法及Log-rank检验进行生存分析。以RR和非复发死亡为竞争风险，采用cmprsk包竞争风险模型进行分析，Gray检验比较组间差异。多因素分析采用Cox回归探讨OS、PFS的影响因素。*P*<0.05为差异有统计学意义。

## 结果

1. 临床特征：184例MDS患者中，男111例，女73例，中位年龄42（10～63）岁，76例（41.3％）患者年龄≥45岁。初诊时，患者中位WBC为2.62（0.75～28.11）×10^9^/L，中位HGB 79（34～142）g/L，中位PLT 50（4～446）×10^9^/L。骨髓原始细胞比例中位数为 3.5％（0～19.0％）。86例（46.7％）有染色体核型异常，复杂核型16例，占异常核型的18.6％，所有核型中合并+8核型33例，合并−7/7q−患者20例。176例患者行二代测序检测，151例（85.8％）患者有基因突变，平均基因突变数为1.9±1.3个，中位基因突变数2（0～6）个，20例（11.4％）患者基因突变数≥4个，突变频率较高的基因依次为U2AF1（29.5％）、ASXL1（21.0％）、RUNX1（14.2％）、NRAS（9.7％）、DNMT3A（8.2％）、TP53（6.8％）、ETV6（6.8％）、TET2（5.7％）等（图1）。

**图1:**
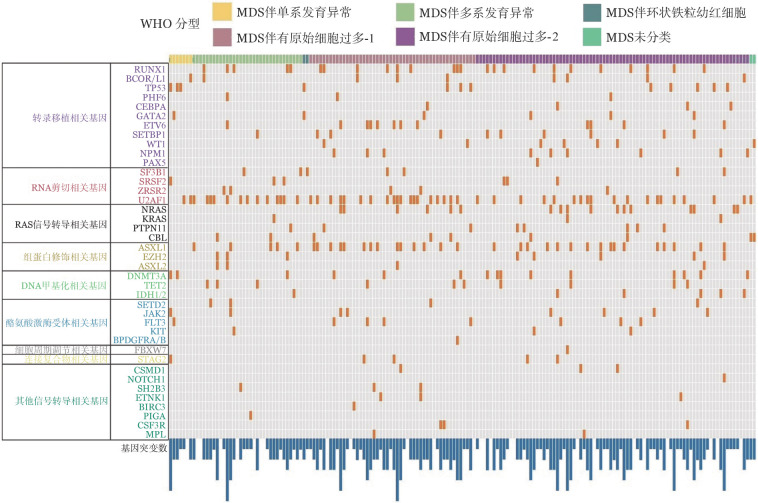
176例骨髓增生异常综合征（MDS）患者二代测序基因图谱

2. 移植结果：181例患者获得重建，1例移植后早期死亡，2例原发性植入失败。获得造血重建的181患者中，所有患者获得粒细胞植入，中位植入时间为12（9～37）d；177例患者获得血小板植入，中位植入时间为14（8～112）d，4例患者未获得血小板植入，为血小板植入不良。在可评价的176例患者中，60例（34.1％）发生aGVHD，中位发生时间为移植后33（8～132）d，aGVHD累积发生率为（31.9±0.1）％，Ⅲ～Ⅳ度aGVHD累积发生率为（11.8±0.1）％，其中不典型2例（1.1％）。在可评价的173例患者中，38例（22.0％）患者发生cGVHD，中位发生时间为移植后143（65～507）d，cGVHD累积发生率为（20.2±0.1）％，其中局限型30例（17.3％），广泛型8例（4.5％）。

3. 生存情况：184例MDS患者中位随访时间21.9（0.5～47.5）个月，140例生存，其中134例为无病生存，44例死亡，死于复发12例，非复发死亡32例，其中6例死于cGVHD，1例死于aGVHD，7例死于器官功能衰竭，3例死于脑卒中，13例死于感染，1例死于肺栓塞，1例死于血栓性微血管病。移植后2年OS、PFS率分别为（75.1±3.4）％、（71.6±3.6）％，累积RR率、非复发死亡率分别为（11.9±0.1）％、（16.5±0.1）％。

4. IPSS-R及WPSS初诊及移植前评分对预后影响：初诊时IPSS-R分组：极低危0例，低危20例，中危43例，高危76例，极高危45例；WPSS分组：极低危2例，低危12例，中危44例，高危101例，极高危25例，IPSS-R以3.5分作为临界点，将≤3.5分归为相对低危组，>3.5分归为相对高危组，初诊时IPSS-R≤3.5分与IPSS-R>3.5分组OS、PFS差异无统计学意义［OS：（82.1±8.1）％对（75.0±4.1）％，*P*＝0.409；PFS：（75.0±15.3）％对（72.4±4.0）％，*P*＝0.724］（图2A、2B）。WPSS以2分作为临界点，初诊时WPSS≤2分与>2分组OS、PFS差异无统计学意义［OS：（72.4±6.4）％对（77.8±4.6）％，*P*＝0.462；PFS：（70.7±9.3）％对（73.8±4.6）％，*P*＝0.726］（图2C、2D）。

**图2:**
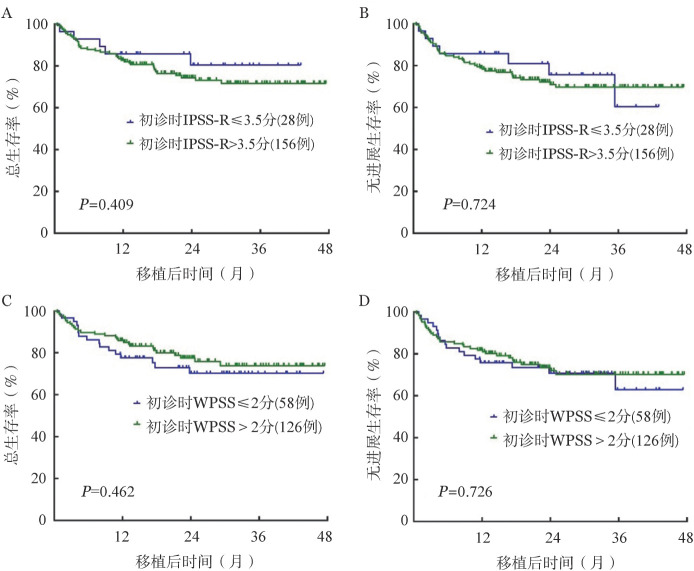
初诊时不同IPSS-R及WPSS评分分组异基因造血干细胞移植治疗后骨髓增生异常综合征患者总生存及无进展生存比较 IPSS-R：修订版国际预后积分系统；WPSS：WHO预后积分系统；A：IPSS-R分组评估总生存；B：IPSS-R分组评估无进展生存；C：WPSS分组评估总生存；D：WPSS分组评估无进展生存

移植前10例患者转化为AML，其余174例患者可进行IPSS-R及WPSS评估，其中移植前IPSS-R分组：极低危15例，低危40例，中危50例，高危38例，极高危31例。WPSS分组：极低危0例，低危5例，中危28例，高危115例，极高危26例。移植前IPSS-R≤3.5分、>3.5分组间OS、PFS差异有统计学意义［OS：（88.6±4.1）％对（65.8±5.3）％，*P*＝0.003；PFS：（87.6±4.2）％对（60.5±5.8）％，*P*＝0.002］（图3A、3B）。移植前WPSS≤2分与>2分组间OS、PFS差异无统计学意义［OS：（71.5±8.1）％对（75.3±4.2）％，*P*＝0.584；PFS：（69.0±8.2）％对（71.2±4.7）％，*P*＝0.565］（图3C、3D）。

**图3:**
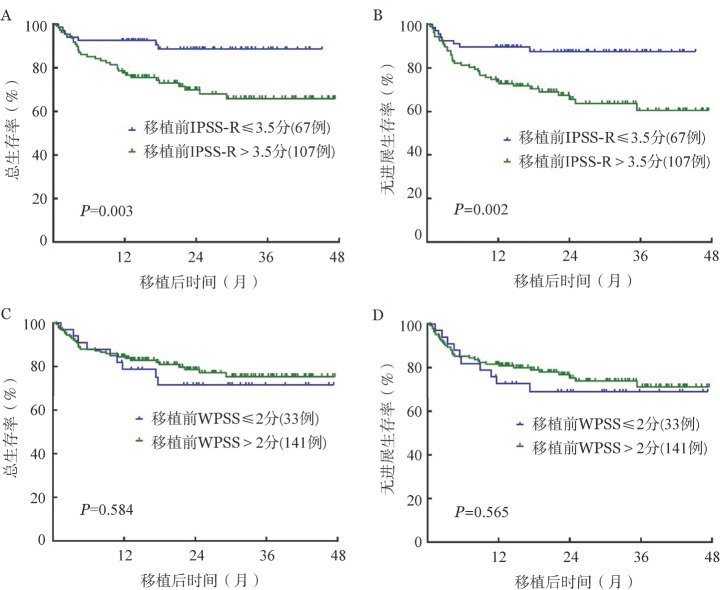
移植前不同IPSS-R及WPSS评分分组异基因造血干细胞移植治疗后骨髓增生异常综合征患者总生存及无进展生存比较 IPSS-R：修订版国际预后积分系统；WPSS：WHO预后积分系统；A：IPSS-R分组评估总生存；B：IPSS-R分组评估无进展生存；C：WPSS分组评估总生存；D：WPSS分组评估无进展生存

移植前IPSS-R较初诊时相比改善组有99例（56.9％），未改善组有75例（43.1％），移植前IPSS-R较初诊时相比改善组OS、PFS显著优于未改善组［OS：（83.8±4.6）％对（69.3±5.8）％，*P*＝0.027；PFS：（82.8±4.4）％对（64.0±7.2）％，*P*＝0.006］（图4）。

**图4:**
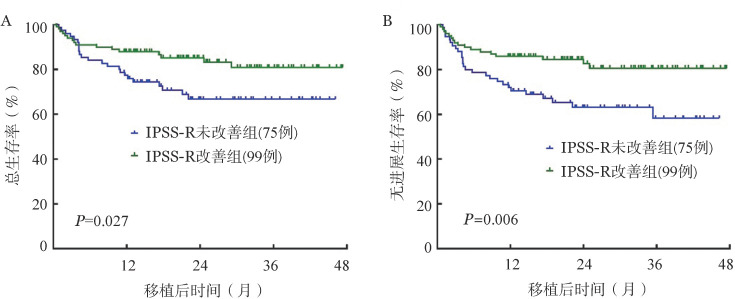
移植前修订版国际预后积分系统（IPSS-R）较初诊时改善与否异基因造血干细胞移植治疗后骨髓增生异常综合征患者总生存（A）及无进展生存（B）比较

5. 预后因素分析：单因素分析显示，患者性别、诊断、供者来源等均不影响OS和PFS。对于MDS-EB2患者而言，移植前接受HMA治疗不影响移植预后。年龄>45岁（*P*＝0.030）、TP53突变（*P*<0.001）、移植前细胞遗传学差及极差（*P*＝0.005）、移植前IPSS-R>3.5分（*P*＝0.002）为影响OS的预后不良因素。TP53突变（*P*＝0.005）、移植前细胞遗传学差及极差（*P*<0.001）、移植前IPSS-R>3.5分（*P*＝0.002）为影响PFS的预后不良因素（表1）。多因素分析显示：TP53突变（*P*＝0.047，*HR*＝2.460，95％ *CI* 1.014～5.971）和移植前IPSS-R>3.5分（*P*＝0.021，*HR*＝2.510，95％ *CI* 1.151～5.476）是影响OS的独立不良预后因素，移植前细胞遗传学差及极差（*P*＝0.008，*HR*＝2.765，95％ *CI* 1.305～5.856）和移植前IPSS-R>3.5分（*P*＝0.017，*HR*＝2.457，95％ *CI* 1.175～5.141）是影响PFS的独立不良预后因素。

**表1:** 影响骨髓增生异常综合征（MDS）患者allo-HSCT后总生存及无进展生存的单因素分析

临床参数	总生存		无进展生存	
	*HR*（95％*CI*）	*P*值	*HR*（95％*CI*）	*P*值
年龄（≤45岁/>45岁）	1.904（1.038～3.492）	0.030	1.582（0.895～2.794）	0.101
性别（男/女）	1.405（0.768～2.570）	0.291	1.432（0.814～2.521）	0.233
诊断		0.920		0.953
其他（参照）	1		1	
MDS-EB1	1.180（0.518～2.690）		0.987（0.463～2.109）	
MDS-EB2	1.076（0.514～2.252）		1.093（0.538～2.220）	
基因突变数（<4个/≥4个）	1.381（0.521～3.663）	0.462	1.183（0.475～2.947）	0.699
TP53突变	3.599（0.929～13.94）	<0.001	3.003（0.857～10.520）	0.005
AXSL1突变	1.068（0.504～2.264）	0.861	0.893（0.443～1.798）	0.455
RUNX1突变	0.734（0.319～1.692）	0.515	0.776（0.355～1.694）	0.560
移植前骨髓原始细胞（≤5％/>5％）	1.785（0.936～3.404）	0.053	1.672（0.914～3.058）	0.069
移植前细胞遗传学（极好+好+中等/差+极差）	3.005（1.065～8.483）	0.005	4.258（1.496～12.12）	<0.001
移植前IPSS-R（≤3.5分/>3.5分）	3.376（1.842～6.187）	0.002	3.131（1.717～5.708）	0.002
移植前是否桥接HMA治疗^a^	2.385（0.946～6.014）	0.088	1.969（0.801～4.846）	0.161
HLA相合	1.142（0.630～2.071）	0.664	1.234（0.707～2.155）	0.464

注：allo-HSCT：异基因造血干细胞移植；MDS-EB：MDS伴有原始细胞过多；IPSS-R：修订版国际预后积分系统；HMA：去甲基化药物。^a^在84例MDS-EB2患者中进行分析

## 讨论

自1997年Greenberg等[2]提出IPSS以来，其被广泛应用于MDS患者的预后分层中。然而，IPSS仅可用于原发初诊的MDS患者，无法在疾病演化中动态监测，同时中危型细胞遗传学规定过于简单，血细胞减少按系列数积分，忽视了严重程度带来的影响，为提高预测能力及适用性，研究者提出了WPSS及IPSS-R[3]–[4]，两者都被证实可动态监测病程中接受来那度胺或HMA的患者，WPSS包括了贫血的严重程度，IPSS-R纳入了最新的细胞遗传学分类、细化了血细胞及骨髓原始细胞比例减少的程度，相比IPSS具有更好的预后评估价值[12]–[13]。

通常情况下，IPSS-R或WPSS分层高危或极高危的患者首选allo-HSCT，低危或中危伴有高危基因突变如TP53、ASXL1、RUNX1、EZH2、ETV6等，或是合并治疗无法纠正的血细胞严重减少及严重输血依赖时也应选择allo-HSCT[7],[14]。已有国外研究者对WPSS及IPSS-R是否可预测MDS移植患者预后进行了探讨，2008年一项来自GITMO的研究，采用WPSS对行allo-HSCT的原发性MDS患者进行危险分层，证实WPSS是预测MDS移植患者预后的有力指标[5]。在另一项单中心研究中，Yahng等[10]在MDS移植患者去甲基化治疗时及移植前行IPSS-R评分，两个不同时间点IPSS-R均可预测患者生存。

本研究探讨了WPSS及IPSS-R在中国MDS移植患者中的预后价值，我们纳入了184例接受allo-HSCT的MDS患者，在初诊时及移植前分别进行WPSS及IPSS-R评分。根据WPSS进行分层，无论是初诊还是移植前相对低危组的生存率反而略低于相对高危组，甚至极低危及低危组的死亡率高于其他组，与国外报道有差异，可能是由于WPSS未细化细胞遗传学分类，忽视了白细胞及血小板对预后的影响，导致区分度偏低，因此WPSS对于本研究接受allo-HSCT治疗的MDS患者并没有显著的预后意义。同样，根据IPSS-R分层，初诊时相对低危组与相对高危组生存率无统计学意义，在相对低危组的28例患者中，23例患者在移植前评分未有改善，13例患者转化为相对高危组，其中5例患者最终死亡，有3例患者伴有高危基因突变，因此，在初诊时单纯的应用IPSS-R亦不能预测移植患者的预后。Hou等[15]基于中国MDS患者数据，开发了一种集年龄、IPSS-R及5个基因突变（TP53、CBL、IDH2、DNMT3A、ASXL1）为一体的预后模型，在368例MDS患者中获得了验证，证实其在OS及无白血病生存上的预后评估效能优于IPSS-R，但是该模型能否应用于移植患者仍需进一步验证。本研究中TP53突变为独立的预后不良因素，与既往文献报道一致[16]–[19]，是否可将TP53突变与IPSS-R结合，建立一种适用于移植患者的新预后模型，仍需进一步探索。

移植前重新对该组患者进行IPSS-R评分，移植前相对低危组患者OS、PFS显著优于相对高危组，在纳入年龄、移植前骨髓原始细胞数、细胞遗传学分类、TP53突变的多因素分析中，移植前IPSS-R仍为独立的预后不良因素，提示移植前再次监测IPSS-R是预测MDS移植患者预后的有效手段，移植前IPSS-R的预后评估价值高于初诊时。我们进一步比较了初诊时与移植前IPSS-R的变化情况，发现移植前IPSS-R较初诊时改善组OS、PFS显著优于未改善组，提示移植前IPSS-R的改善可以带来更好的预后。以往报道显示染色体核型显著影响患者预后[9]，尤其单体核型患者复发率高[20]。本研究中移植前细胞遗传学预后差/极差组患者PFS率显著低于极好/好/中等组，这提示我们对于此类患者移植后需加强监测。

综上所述，本研究结果显示移植前IPSS-R是影响患者OS及PFS的独立危险因素，对移植前的MDS患者进行IPSS-R评分是预测移植后生存的有效手段，移植前IPSS-R的改善可以带来更好的预后。TP53突变及移植前细胞遗传学为影响MDS移植患者预后的独立危险因素。因此，建立基因突变数据整合到预后积分系统的预后评分模型将是进一步研究方向。
